# Hypercalcemia of Malignancy: An Emergency Medicine Simulation

**DOI:** 10.7759/cureus.1847

**Published:** 2017-11-15

**Authors:** Raman Sohi, Gillian Sheppard

**Affiliations:** 1 Faculty of Medicine, Memorial University of Newfoundland

**Keywords:** hypercalcemia, simulation, hypercalcemia of malignancy, emergency medicine, oncology, cancer, palliative care, breaking bad news

## Abstract

Hypercalcemia is a poor prognostic factor associated with malignancy. The signs and symptoms of hypercalcemia that the patients present to the emergency department are vague and often overlap with the general symptoms of cancer itself or the adverse effects of the chemotherapy. Given that the development of hypercalcemia of malignancy can present with imminent danger to the patient and is a treatable condition, emergency physicians should know how to recognize and treat it. It also marks a time at which discussions regarding plans of care should be initiated with the patients. In this report, we describe a simulation case that can be used to train emergency medicine residents to both recognize and treat hypercalcemia of malignancy and to initiate the discussion of goals of care.

## Introduction

Hypercalcemia associated with malignancy is relatively common, occurring in up to 20-30% of the patients with cancer [1]. The leading cause of hypercalcemia in hospitalized patients is hypercalcemia associated with cancer [2]. This condition can occur in patients with both solid and hematologic malignancies, with the most common malignancies being breast cancer, lung cancer, multiple myeloma, and renal cell carcinoma [2-3].

The main pathogenesis of the hypercalcemia is increased osteoclastic bone resorption, however, the renal tubular resorption and intestinal absorption of calcium can contribute as well [1-2]. The bone resorption can occur due to increased tumor secretion of parathyroid hormone-related protein (PTHrP), osteolysis secondary to bone metastasis, or tumor production of calcitriol [4-5].

The signs and symptoms of hypercalcemia are often nonspecific, such as fatigue, nausea, constipation, and confusion [2], making the diagnosis challenging. These clinical features often mimic general symptoms of underlying cancer itself or adverse effects from the treatments such as chemotherapy. This simulation was created to assist emergency medicine residents to consider hypercalcemia when oncology patients present with acute changes in symptoms.

The development of hypercalcemia of malignancy indicates a poor prognosis for the patients with cancer. It is reported that 80% of the patients die within a year [2] and 50% die within 30 days of presentation [1]. For this reason, when a diagnosis of hypercalcemia is made in the patient population, a discussion regarding the goals of care needs to be initiated. There is a lack of formal palliative care training in many emergency medicine residency programs [6]. Case-based simulation, bedside teaching have been shown to be the most effective educational methods to provide palliative care teaching to these residents [6]. Therefore, this simulation case was also created to provide emergency medicine residents with effective methods of initiating palliative care discussions.

The learning objectives covered by this simulation case are:

1. Recognize acute illness in an oncology patient

2. Recognize the signs and symptoms of hypercalcemia in an oncology patient and consider the differential diagnosis

3. Order appropriate labs and diagnostic imaging

4. Initiate treatment for hypercalcemia of malignancy

5. Deliver bad news and initiate discussion around goals of care.

## Technical report

Context

The simulation session was conducted in the Clinical Learning and Simulation Centre (CLSC) at the Faculty of Medicine, Memorial University. It can be, however, performed in any medical school or hospital-based simulation lab, or in an emergency department. The simulation was conducted to train emergency medicine residents; however, other physicians, medical students, and the nurses may benefit from the case.

Inputs

This simulation case requires standardized patients (SPs) playing the roles of the patient and her husband, and another SP playing the role of the emergency room nurse. Instructors will be present to control the patient’s vitals and to observe the scenario unfold. This case is preferably performed where there is an examining table, a cardiac monitor, an intravenous (IV) setup, and a screen to display X-rays to preserve the reality of the scenario.

**Figure 1:**
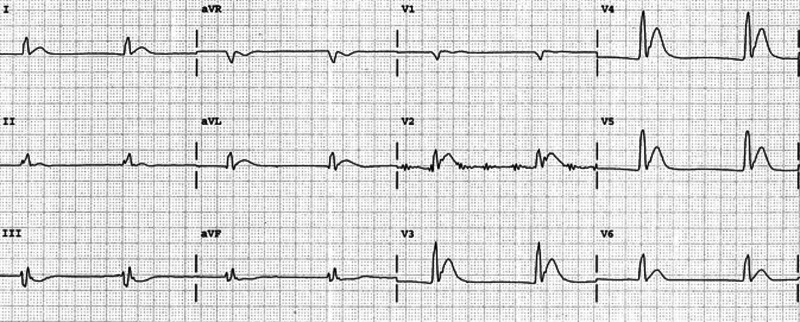
The electrocardiogram showing marked shortening of the QT interval (260 ms) from hypercalcemia. Source: Life in the fastlane - http://lifeinthefastlane.com/ecg-library/basics/hypercalcaemia/

**Figure 2:**
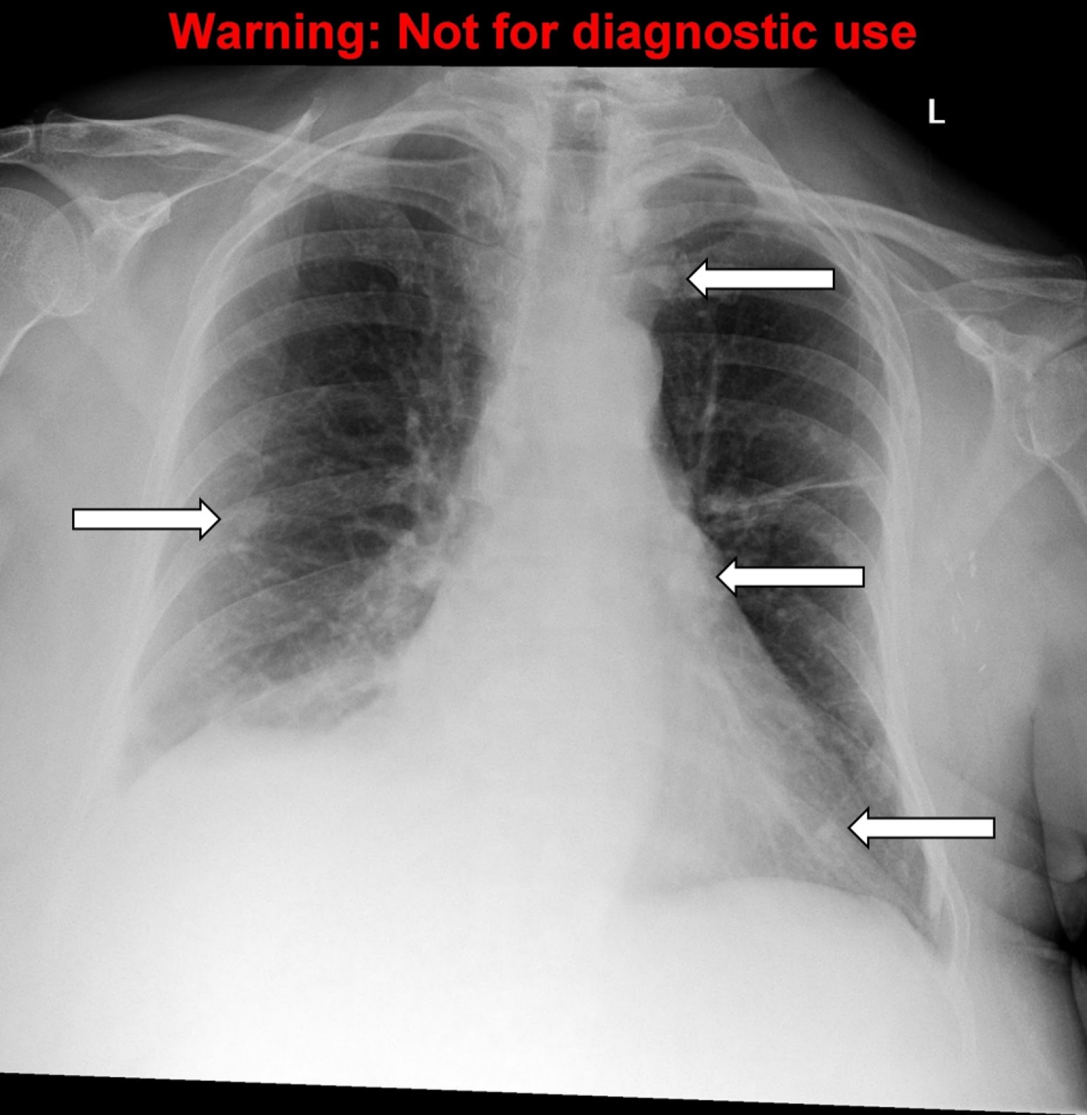
The chest X-ray, anteroposterior (AP) view, demonstrating bone metastasis. Source: A Dixit

**Figure 3:**
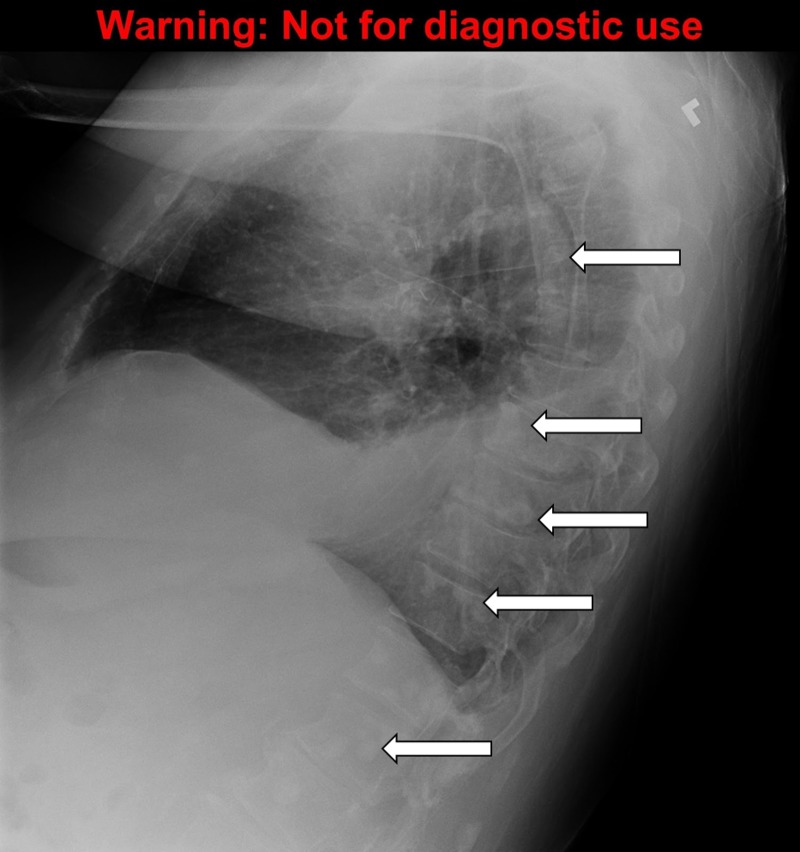
The chest X-ray, lateral view demonstrating bone metastasis. Source: A Dixit

**Figure 4:**
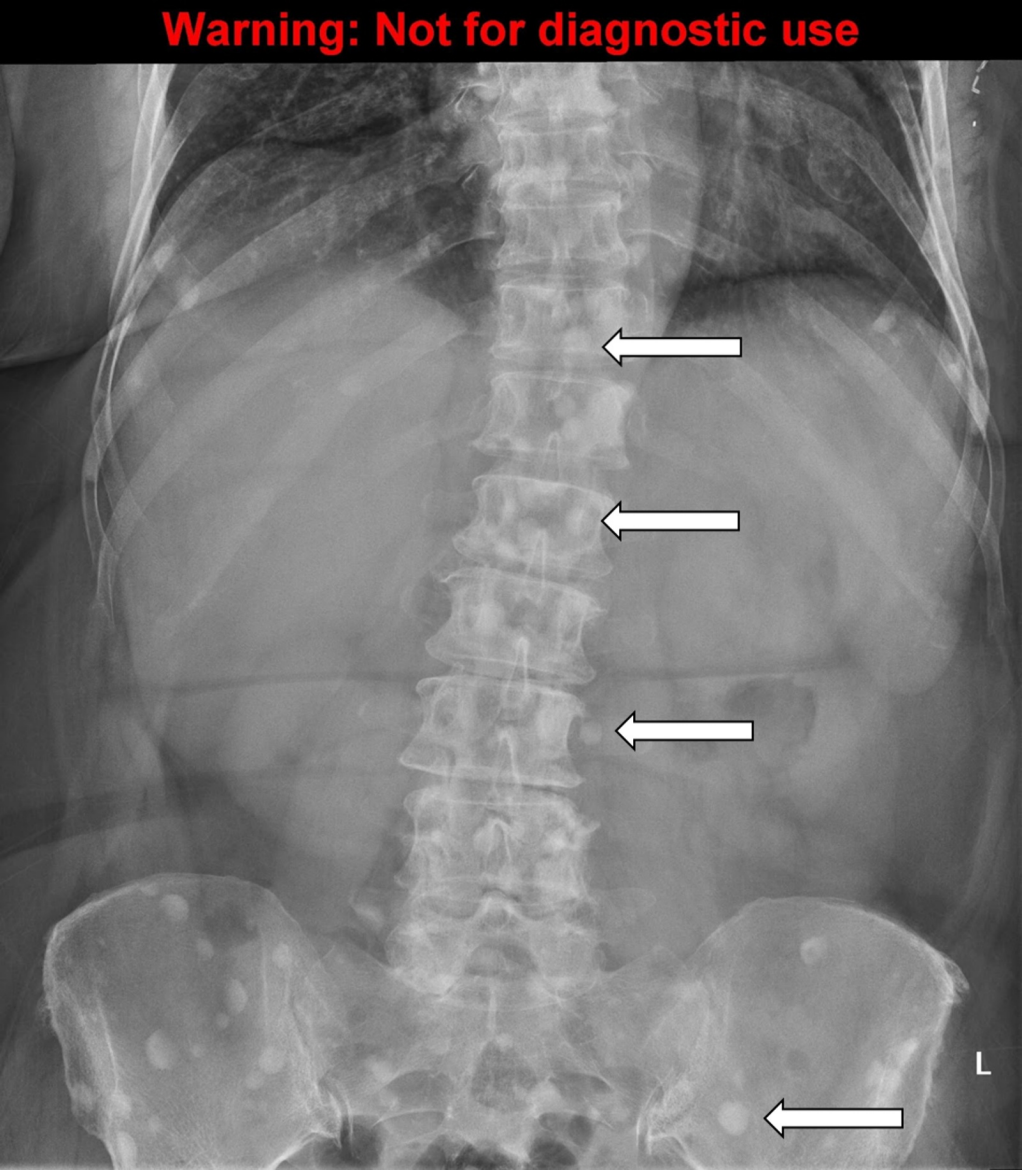
The abdominal X-ray demonstrating bone metastasis. Source: A Dixit

**Figure 5:**
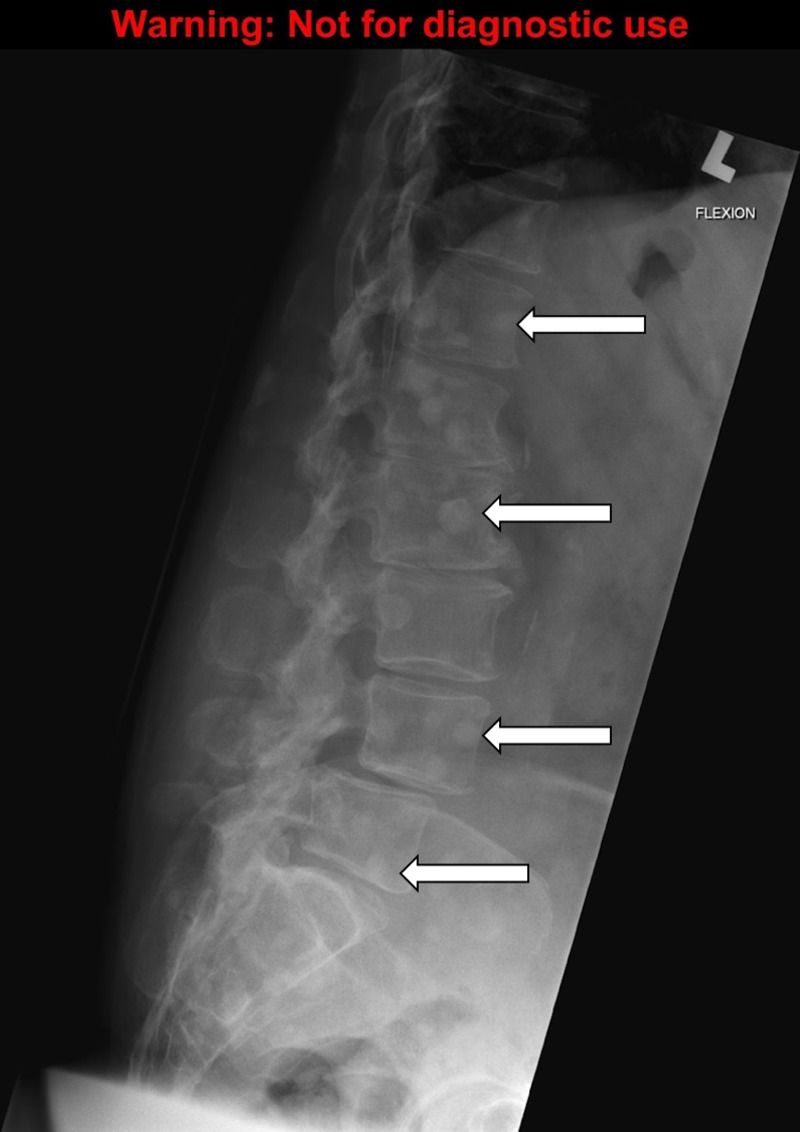
The lumbar X-ray demonstrating bone metastasis. Source: A Dixit

A detailed stepwise scenario template (Table 1), as well as a script for each SP (Appendices A-C) were created. An electrocardiogram (ECG) tracing of hypercalcemia (Figure 1) and multiple X-rays demonstrating bone metastasis (Figures 2-5) were also obtained. Prior to the training session with the residents, a mock scenario was completed with the SPs and an instructor to ensure the learning objectives would be appropriately demonstrated by the case.

**Table 1:** A stepwise, detailed scenario template to be submitted to the simulation lab and the standardized patient coordinators, who then train the standardized patients and supply the necessary materials for the case Legend: L4-5 – 4^th^ and 5^th^ lumbar vertebrae, ECG – electrocardiogram, IV – intravenous, CBC – complete blood count, BUN – blood urea nitrogen, PTH – parathyroid hormone, TSH – thyroid stimulating hormone, CXR – chest X-ray, CT – computed tomography, AST – aspartate transaminase, ALT – alanine transaminase, ALP – alkaline phosphatase, INR – international normalized ratio, PTT – partial thromboplastin time, SC – subcutaneously, SPIKES: S- setting up the interview (ensure privacy, involve family, make a connection with the patient and avoid interruptions), P- assess the patient’s perception (open-ended questions and correct misinformation), I- obtain the patient’s invitation (information disclosure, does the patient want all information?), K- giving knowledge (warn the patient of bad news, give facts in small chunks and avoid bluntness), E- address the patient’s emotions (observe and identify), S- strategy and summary (the treatment plan and the goals of care).

Pre-Scenario:		
You are a family physician in a rural emergency department. A 59-year-old female with stage 4 metastatic breast cancer presents to you confused and complaining of increased nausea and fatigue over the past week. She has had a double mastectomy with chemotherapy five years ago for Stage 2 breast cancer. However, six months ago, she had a recurrence and is once again being treated with chemotherapy. The patient has not been connected with any pain or symptom management teams.		
History: Over past week, the patient was more confused, complaining of nausea and fatigue.		
Allergies	No known allergies	
Medications	Hydrochlorothiazide, ondansetron, dexamethasone, cyclophosphamide, doxorubicin, 5-Fluorouracil (FAC)	
Past medical history	Stage 2 breast cancer, unilateral modified radical mastectomy, hypertension, cesarean section, appendectomy	
Social history	Married, two children (18 & 20 years old), previous smoker (10 pack-year history), no alcohol. Prior to cancer, worked as a teacher.	
Review of systems	General – weight loss (~5-10 lbs over past few weeks), lethargic, decreased appetite, no fever/chills; eyes – no visual changes; ears, nose, throat – no hearing changes, no sore throat; cardiovascular – no chest pain, no palpitations, no shortness of breath, no peripheral edema; respiratory – no cough, no shortness of breath, no wheezing; gastrointestinal – nausea, decreased appetite, no vomiting, no abdominal pain, no change in bowel movements; genitourinary – no change in urination; musculoskeletal – pain in the chest and lower back; neurologic – drowsiness, dizziness on standing, no headache, no numbness or tingling; skin – no rash	
Physical: Appears thin, lethargic, and cachectic, in some discomfort		
Initial vitals	Temperature 37 (oral), heart rate 59 (sinus), blood pressure 85/50, respiratory rate 18	
Head, eyes, ears, nose, throat	Pale, dry mucous membranes, no thyroid nodules on palpation	
Central nervous system	Alert, oriented to person and place – not oriented to time, Glasgow Coma Scale 14, cranial nerves I-XII intact, reflexes normal in upper and lower limbs	
Cardiovascular system	Heart sounds normal, slow heart rate, jugular venous pressure flat (difficult to see), pain to palpation across chest wall (rib metastasis), mastectomy scar on the chest	
Respiratory system	Normal breath sounds, no wheezes, no crackles, mastectomy scar on the chest	
Abdomen	Cesarean section scar, appendectomy scar, otherwise benign	
Musculoskeletal System	Lower back tenderness (L4-5 region) (vertebral metastasis)	
Expected actions (immediate)		
Assign to bed space with cardiac monitor, connect to cardiac monitor and oxygen saturation probe		
Check bedside glucose		
Obtain 12-lead ECG		
Start 2 large bore IVs, order IV normal saline 1 L bolus		
Order labs (CBC, electrolytes, glucose, BUN, creatinine, calcium, magnesium, phosphate, albumin, liver function panel, PTH, TSH, toxicology screen, urinalysis)		
Order diagnostic imaging (CXR, the abdominal series, lumbar series, the CT head scan)		
Engage family member (husband)		
Objective 1: Recognize acute illness in an oncology patient		
Stage 1: Initial Assessment		
Stage	Findings	Expected action
Recognize acute presentation	Confusion, nausea and lethargy x one week	Get new set of vitals, order labs
Get new set of vitals	Same as above	Start two large bore IVs, order IV normal saline 1 L bolus
With fluid bolus	Heart rate 59, blood pressure 90/60	IV normal saline 1 L bolus
Without fluid bolus	Heart rate 59, blood pressure 80/48	
Assess for pain	Pain to palpation across the chest wall, lower back tenderness	Pain control should be given (IV Ketorolac or Morphine) + Anti-emetic (IV dimenhydrinate 25-50 mg, ondansetron 4-8 mg, or metoclopramide 10 mg) Order CXR, abdominal series, lumbar series
Objective 2: Recognize the signs and symptoms of hypercalcemia in an oncology patient and consider the differential diagnosis Objective 3: Order appropriate labs and diagnostic imaging		
Stage 2: Recognize cause of symptoms		
Results of ordered labs	Hemoglobin 125, Hematocrit 0.39, Platelets 200, White blood cell count 5.0, Sodium 135, Chloride 100, Potassium 4.3, Calcium 3.5 (corrected for albumin = 3.6), Magnesium 0.9, Phosphate 0.98, Glucose 4.8, BUN 5.0, Creatinine 79, Albumin 35, AST 35, ALT 30, ALP 309, Total bilirubin 15, INR 1.2, PTT 32, PTH <2.5, TSH 2.5, toxicology screen negative	Recognize elevated calcium level. Recognize acute presentation may suggest hypercalcemia of malignancy. Consult patient for the internal medicine.
Objective 4: Initiate treatment for hypercalcemia of malignancy		
Stage 3: Initiation of the treatment		
Initiate normal saline rehydration		200-500 mL/h IV
Initiate Bisphosphonate (Zoledronate)		4-8 mg IV over 15 mins
Initiate calcitonin	May or may not be initiated	2-8 units/kg SC every six-12 hours (52 kg = 104-416 units SC every six-12 hours) or 100 units SC three times a day
Discontinue hydrochlorothiazide	Hydrochlorothiazide can contribute to hypercalcemia	Change to another anti-hypertensive
Objective 5: Deliver bad news and initiate discussion around goals of care		
Results of CXR, abdominal & lumbar series	Extensive bone metastasis to ribs, lumbar vertebrae, pelvis present	Discuss with the patient
Use strategy (such as “six steps”, SPIKES, CLASS) to explain cancer has spread to bones and is incurable		Assess patient’s understanding of the disease. Assess patient’s goals of care. Is there an advance care directive?
Disposition		Consult internal medicine

 

 

Process

The resident is given pre-scenario information, along with initial vitals prior to entering the scene. Upon entering, the patient is already in a hospital gown and connected to a cardiac monitor. The emergency room nurse and the patient’s husband are also present in the room. The residents proceed with their assessment.

Product

The scenario was completed as a formative exercise for the residents at Memorial University. However, there is the potential to have it formally evaluated using the five objectives that the case is expected to achieve.

Pre-briefing

A pre-briefing was held with the residents before they began the case. The instructors involved in the case were identified. The residents participating in the case were determined; in this case, two residents completed the simulation. The fiction contract was reviewed, which encourages residents and instructors to proceed as if the case was real, while concurrently identifying it is not real. This provides a safe environment for residents to learn. 

Case

This simulation takes place in a community hospital. A 59-year-old female is brought to the emergency department by her husband. She has a history of breast cancer and presents with worsening symptoms over the past week. She complains of increased nausea and fatigue, along with feeling confused. 

Debriefing

Upon conclusion of the scenario, the instructor completes a formal debriefing exercise with the residents. Each resident is given an opportunity to safely express his or her feelings and reflections of the case, the events of the case are reviewed, and the SPs provide feedback. The debriefing with a good judgment approach can be used to create a safe environment where the instructor can provide feedback without being penalized, or without eliciting a defensive response from the residents [7]. This method involves the instructor clarifying actions of the residents that they may question, by assessing the resident’s reasons for the action. This avoids a single “right” or “wrong” answer, and instead maintains respect for the resident as a capable physician. An example dialogue could include, “During the case, I noticed you did (action). Can you help me understand why you chose to do (action) at that point?”. The debriefing with good judgment approach helps residents integrate their insights and experiences so that they can improve their performance when a similar situation presents in the future [7].

Post-scenario didactics

After the debriefing, a didactic session is held to focus on knowledge gaps identified throughout the simulation case and debriefing session. The objectives are used to strengthen and solidify the knowledge learned from the simulation exercise.

Objective 1

To recognize acute illness in an oncology patient. Recognizing acute illness in the patients with cancer can be difficult, as they are often quite sick at baseline. In addition to assessing the patient’s new or worsening symptoms, it is also helpful to assess the patient’s functional status. The Palliative Performance Scale (PPS) is an 11-point scale (Appendix D) describing the patient’s level of ambulation, level of activity, evidence of the disease, ability to perform self-care, nutritional intake, and level of consciousness [8-9].

Objective 2

To recognize the signs and symptoms of hypercalcemia in an oncology patient and consider the differential diagnosis. These patients often present with nonspecific clinical features such as fatigue, nausea, constipation, and confusion, making diagnosis challenging. Severe symptoms that are important for the emergency physician to recognize include seizures, cardiovascular collapse, and coma [1-2]. The differential diagnosis will depend on the patient’s symptoms. In this, the patient with nausea, fatigue, and confusion, some diagnoses to consider are adverse effects of chemotherapy, the progression of breast cancer, dehydration, hypo/hyperglycemia, electrolyte disturbances, delirium, and gastrointestinal disorders or infections.

Objective 3

To order appropriate labs and diagnostic imaging. The symptomatic cancer patients should always have plasma calcium measured. The calcium levels can be classified as mild (2.6-2.9 mmol/L), moderate (3.0-3.4 mmol/L), and severe (≥ 3.5 mmol/L) [1]. The formula to correct calcium for the albumin level should be reviewed and is: measured calcium (mmol/L) + ( [40 – albumin (g/L) ] x 0.02) [2]. The levels of parathyroid hormone (PTH) should be undetectable or extremely low unless primary hyperparathyroidism is also present. For diagnostic imaging, a chest x-ray, the abdominal and lumbar series are reasonable to investigate bone pain and the possibility of bone metastasis, looking for lytic or sclerotic lesions. A computed tomography (CT) scan of the head may also be reasonable to investigate the new onset of confusion and weakness and to rule out brain metastasis.

Objective 4

To initiate treatment for hypercalcemia of malignancy. The reduction of tumor mass usually corrects hypercalcemia, but this is not always possible, as is the case in this scenario with metastatic disease [10]. The mainstay of the treatment of hypercalcemia is hydration with normal saline and IV bisphosphonates (zoledronate or pamidronate). The IV bisphosphonates should be started right away, as they take 12 hours for onset, with full effect reached by four-seven days. They block osteoclast activity and reduce bone pain. The zoledronate requires 15-minute infusions and pamidronate requires two-four hour infusions. Both need to be dose-adjusted for the renal dysfunction. The calcitonin works rapidly, but the effect is short-lived as the patients develop tolerance in two-three days. It may be started initially, especially in the patients with severe symptoms, while waiting for the bisphosphonate to take effect. The calcitonin is administered subcutaneously. It used to be standard practice to administer loop diuretics (furosemide) in hypercalcemia; however, there is a lack of evidence for effectiveness [3]. Thiazide diuretics, like the hydrochlorothiazide that this patient takes for hypertension increases renal calcium resorption, so these medications should be discontinued/switched to an alternative [11].

Objective 5

To deliver bad news and initiate discussion around the goals of care. Hypercalcemia of malignancy has a poor prognosis and is associated with advanced disease. When diagnosed, the physicians should discuss the goals of care and advance care directives with the patient and family. Strategies such as SPIKES are helpful: S- setting up the interview (ensure privacy, involve family, make a connection with the patient and avoid interruptions), P- assess the patient’s perception (open-ended questions and correct misinformation), I- obtain the patient’s invitation (information disclosure, does the patient want all information?), K- giving knowledge (warn the patient of bad news, give facts in small chunks and avoid bluntness), E- address the patient’s emotions (observe and identify), S- strategy and summary (the treatment plan and the goals of care) [12]. A referral to palliative care may be appropriate.

## Discussion

Teaching emergency medicine residents how to recognize and manage hypercalcemia of malignancy using case-based simulation is a valuable teaching tool. Hypercalcemia of malignancy is an indicator of poor prognosis and the recognition and initiation of discussions regarding goals of care are important.

The learning objectives covered by this simulation case include:

1. Recognize acute illness in an oncology patient 2. Recognize the signs and symptoms of hypercalcemia in an oncology patient and consider the differential diagnosis 3. Order appropriate labs and diagnostic imaging 4. Initiate treatment for the hypercalcemia of malignancy 5. Deliver bad news and initiate discussion around the goals of care.

This case scenario is outlined in a stepwise approach for replication. It is adaptable and can be altered based on the needs of the trainees, level of training of those involved, and the resources available at the site.

In addition to the simulation case, the residents will also benefit from the debriefing session and post-scenario didactics. These sessions allow residents to reflect on their experience and allow instructors to identify knowledge gaps and provide necessary teaching. Collectively, this will provide the emergency medicine residents with the knowledge and confidence to provide appropriate care to the patients with hypercalcemia of malignancy in the future.

## Conclusions

Hypercalcemia of malignancy can be challenging to recognize, and once diagnosed will involve a no doubt difficult discussion with the patient regarding their condition. Therefore, providing the emergency medicine residents with a case-based simulation training session allows for a controlled and safe environment to learn the techniques of approaching this situation. We have presented a simulation case of hypercalcemia of malignancy using a stepwise algorithm, along with an approach to debriefing and providing didactic teaching.

## Appendices

Appendix A

Script for standardized patient (SP)

Opening Statement

“Oh, I’m not feeling well, doctor. I feel like I’m going to throw up.”

Chief Complaint

You are a 59-year-old female who is presented to the emergency department in Clarenville, Newfoundland due to increasing nausea and almost fainting while going to the bathroom.

History of the Present Illness

For the past week, you have been feeling increasingly nauseated and tired/fatigued. You have not vomited; however, nausea has been getting worse and your appetite has decreased as well.

You have also been experiencing a new onset of lower back pain over the past week. The pain is an 8/10 dull ache with no radiation, and you are having very little relief with Tylenol and Advil. You started noticing the back pain about a week ago and it has been gradually progressing. If asked to point to the pain, point to lower back around the level of the dimples.

Past Medical History

You have a history of breast cancer. You were diagnosed with Stage 2 breast cancer in 2011, which was treated with a unilateral modified radical mastectomy and chemotherapy. However, six months ago cancer returned and your oncologist told you it was Stage 4 and has started you on chemotherapy again. Your last chemotherapy dose was two weeks ago.

Other medical history includes diagnosis of hypertension in 2000, which is being treated with medication.

The surgical history includes the mastectomy in 2011, a cesarean section in 1998, and an appendectomy in 1969.

Medications

Cyclophosphamide, doxorubicin, 5-Fluorouracil (FAC), this is the chemotherapy; the last dose was two weeks ago

Ondansetron (Zofran) 8 mg twice a day as directed for prevention of nausea and vomiting related to chemotherapy

Dexamethasone 8 mg as directed before chemotherapy for the prevention of nausea and vomiting related to chemotherapy

Hydrochlorothiazide 25 mg once daily for hypertension

Family History

Your parents are alive and well. They live on the west coast of Newfoundland in Pasadena (five hours away). Your mother is 80 years old, and father is 83 years old. They do not have significant health issues that you are aware of. You have one sister who is 57 years old living in Sudbury, Ontario. She is alive and well with no significant health issues. Your grandparents have passed away; the cause of death is unknown. You have two children; an 18-year-old female and a 20-year-old male. Both are healthy with no significant health issues.

Social History

The previous smoker, you smoked one pack/day for 10 years; you quit in 2011 after your diagnosis of cancer. You are nondrinker. You are a retired teacher, having retired when you were diagnosed with cancer in 2011. Married, the husband is a teacher and very supportive. He is accompanying you to the hospital right now. You have two children; an 18-year-old female and a 20-year-old male. Your son is studying engineering at Memorial University in St. John’s. Your daughter is graduating high school this year in June.

Acting Instructions

You are to act nauseated, fatigued, and slightly confused. You are aware of your illness and that there is no cure, but you really want to live long enough to witness your daughter’s high school graduation. If asked when the graduation is taking place, you are confused and cannot remember the date and have to ask your husband.

Throughout the encounter, you should ask if you will be able to go home tonight and if you will make it see your daughter graduate. You should also ask for something for your back pain.

Nausea: If specifically asked about nausea, say that it started about a week ago and has been getting worse ever since. It is more bothersome than usual nausea you have with chemotherapy, and it is not responding well to medication. You have not vomited as a result of nausea. Over this past week, your appetite has also decreased.

Pain: If specifically asked about the pain, it is about the dull character, located in the lower back (if asked to point to the pain, point to lower back around the level of the dimples). You started noticing the pain about a week ago and it has been gradually increasing, the pain is not radiating or moving anywhere else, and you can rate it as 8/10 if asked. You have not experienced pain like this before, and it has woken you up at night. You have not had any recent injuries or falls. There is not a certain position or movement that worsens the pain or relieves it. You have tried taking Tylenol and Advil with minimal relief.

If the resident does a physical exam on you and palpates your chest, you are to report tenderness on your chest. You will also feel tenderness on palpation of the lower back.

You should ask for something for your back pain if the resident does not start any pain medication.

Fatigue: If specifically asked about fatigue/tiredness, you have been generally unwell and tired since the diagnosis of the progression of your cancer; however, over the last week, you are feeling increasingly fatigued. You have decreased energy and have not been doing any work around the house like you normally would (washing dishes, watching television, etc.). You have been feeling drowsy and have been sleeping a lot. You have not been light-headed or fainted, and you are not experiencing fevers or night sweats.

Confusion: If asked if you’ve been feeling confused, you can admit that you have had trouble remembering details lately and need to ask your husband to clarify things. This has been occurring specifically over this past week.

Affect: You are very tired and have little energy. You feel nauseous, so you aren’t overly talkative but are able to answer questions.

Appearance and clothing: You are thin, cachectic, fatigued, and pale. You are dressed in a hospital gown.

Feelings: You have been having a rough week and are feeling quite ill. You understand you are unwell and your cancer is incurable, but you want to go home soon and continue with the chemotherapy. You are worried about your daughter’s high school graduation in June. You are also worried about your family’s financial situation since you are no longer able to work.

Ideas: You are aware of your incurable condition but are hoping that the chemotherapy will help to keep you alive long enough to see your daughter graduate in June.

Function: You have not been functioning the way you normally do over this past week. You have been increasingly fatigued and drowsy, so you have been sleeping a lot more than normal. You usually walk around the house and do some simple chores such as washing the dishes and basic cleaning, but you have not been able to do these tasks this past week. You have still maintained your ability to clean and dress.

Expectations: You think there may be something wrong since you’ve been feeling more unwell over the past week than normal. You assume some adjustments may be made, but you expect to be sent home to continue your chemotherapy, which you believe will help keep you alive to see your daughter graduate. You know your cancer is incurable at this stage.

Palliative Care: The resident should recognize your condition and realize you are in an advanced stage of the disease. He/she should admit you and initiate a conversation about palliative care or end-of-life care. Your perception of your illness is that you know it is incurable, but you believe you have more time than you actually do. When the resident breaks the bad news, you can express sadness and be upset, especially when you realize you may not make it see your daughter graduate which was very important to you. Ultimately, the resident may suggest a referral to palliative care and you can agree to this along with your husband’s support.

Appendix B

The script for Standardized Patient (SP), the husbands' role.

Opening Statement

Your wife will open with “Oh, I’m not feeling well, doctor. I feel like I’m going to throw up.”

Chief Complaint

You are a 60-year-old male and you have presented to the emergency department in Clarenville, Newfoundland with your wife who just became quite dizzy while getting up to go to the bathroom and almost fainted. Your wife has not been feeling well for the past week. She seems to be confused and very nauseous.

History of Present Illness of Your Wife

You have noticed your wife has been increasingly confused over the past week, which is not normal for her. She has been repeating questions and having a hard time making decisions throughout this week. She has not been oriented to what time of the day it is and has been sleeping for most of the day. She seems to be very fatigued and drowsy this week and you are concerned about her health, especially since she has cancer.

She has not vomited, but her nausea has been getting worse over the past week and her appetite has decreased as well.

She has also been complaining of a new low back pain over the past week. She is having very little relief with Tylenol and Advil.

Past Medical History of Your Wife

Your wife has a history of breast cancer. She was diagnosed with Stage 2 breast cancer in 2011, which was treated with a unilateral modified radical mastectomy and chemotherapy. However, six months ago cancer returned and her oncologist told you it is Stage 4 and has started her on chemotherapy again. Her last chemotherapy dose was two weeks ago.

Other medical history includes diagnosis of hypertension in 2000, which is being treated with medication.

Surgical history includes the mastectomy in 2011, a cesarean section in 1998, and an appendectomy in 1969.

Medications of Your Wife

Cyclophosphamide, doxorubicin, 5-Fluorouracil (FAC) - this is the chemotherapy; the last dose was two weeks ago

Ondansetron (Zofran) 8 mg twice a day as directed– for prevention of nausea and vomiting related to chemotherapy

Dexamethasone 8 mg as directed before chemotherapy – for prevention of nausea and vomiting related to chemotherapy

Hydrochlorothiazide 25 mg once daily – for hypertension

Social History

You are extremely supportive of your wife and are always there to help her. You help her with work around the house and have taken on many of the household responsibilities. You ensure she takes her medications and accompanies her to all of her appointments. You teach at the school in Clarenville. However, since your wife has been diagnosed with Stage 4 cancer, you are working as a substitute so you can spend more time with her. You have two children, an 18-year-old female, and a 20-year-old male. Your son is studying engineering at Memorial University in St. John’s. Your daughter is graduating high school this year in June.

Acting Instructions

You are a supportive husband to your wife. You are sad that your wife is in this condition and that her cancer has returned. You are genuinely concerned about her well-being, and you are curious as to why she has been so sick this past week. Your wife will be able to answer the questions surrounding her nausea and back pain. You will provide input on her confusion.

You can ask the resident why your wife is so confused.

Confusion: Your wife has been confused over the past week. She has never had any issues with confusion or memory in the past. This week she has been confused about recent events and what time of the day it is. For instance, she suggested it was time to eat lunch when it was actually 10 o'clock at night. She has asked you the same questions repeatedly and needs clarification on details. She has not been making simple decisions like she used to, such as what to eat or what to watch on television. Her long-term memory hasn’t been affected, however.

Fatigue: Your wife has been quite fatigued this week, more so than normal. She has been drowsy and sleeps for a lot of hours in the day. She hasn’t had much energy and has not been very involved with the children, especially this past week. She normally keeps in touch with your son who is living in St. John’s studying engineering, however, she has not called him in a few days.

Nausea: Your wife has been complaining of nausea. She has not had much of an appetite, has decreased her food intake and is losing weight over time.

Effect: You are sad that your wife’s cancer has returned, and you are concerned about your wife and anxious to know what has been causing her to feel so sick this past week.

Clothing: Appearance is appropriate and clothing is casual. No special requirement.

Feeling: You are quite anxious about the recurrence of your wife’s cancer and her current state. You will support her in any way you can, but you feel worried about your family at the same time. You are aware of the incurable state of your wife’s condition, so you are concerned about being a single parent for your children later on. You worry about your family’s financial status from time to time as well.

Ideas and expectations: You know your wife’s condition is incurable, but you hope she will be able to “bounce back” from the chemotherapy like she did before.

Palliative Care: The resident should let you know that your wife is in an advanced stage of the disease. A conversation regarding the end of life care will be initiated. When this bad news is shared, you can express sadness and be upset. Losing your wife, who you have always supported, has finally become a very real possibility. Ultimately, the resident may suggest a referral to palliative care and you and your wife can agree to this suggestion.

Appendix C

Script for Standardized Patient (SP), nurses' role.

Acting Instructions

You will be available to give updates on the patient as the resident proceeds through the station.

When asked for initial vitals:

Temperature 37 (oral), heart rate 59 (sinus), blood pressure 85/50, respiratory rate 18

The neurological assessment is as follows:

Glasgow Coma Scale 14, alert, oriented to person and place, not oriented to time.

If a fluid bolus is given, vitals change:

Heart rate 59, blood pressure 90/60

If fluid bolus is not given, vitals change:

Heart rate 59, blood pressure 80/48

If labs are ordered, these are the results (report only results of tests ordered):

Hemoglobin 125

Hematocrit 0.39

Platelets 200

White blood cell count 5.0

Na (sodium) 135

Cl (chloride) 100

K (potassium) 4.3

Ca (cancer antigen) 3.5

Mg (microalbumin) 0.9

PO_4_^-^ (serum phosphate) 0.98

Glucose 4.8

Blood urea nitrogen (BUN) 5.0

Creatinine 79

Albumin 35

Aspartate aminotransferase (AST) 35

Alanine aminotransferase (ALT) 30

Alkaline phosphatase (ALP) 309

Total bilirubin 15

 International normalized ratio (INR) 1.2

Partial thromboplastin time (PTT) 32

Parathyroid hormone (PTH) <2.5

Thyroid stimulating hormone (TSH) 2.5

The toxicology screen was negative.

The resident may order diagnostic imaging (X-ray, head CT scan, etc). You can agree to these orders. The results may not be available during the interaction.

If the resident asks you to start medications, get the medication name, dose and route. If the resident wants to dose based on weight, report the patient’s weight as 52 kg. You can tell the resident the medication has been started/given.

Appendix D

**Table 2:** The Palliative Performance Scale. PPS - Palliative Performance Scale Source: Lau F, Downing F, Michael G, Lesperance M: A reliability and validity study of the palliative performance scale. BMC Palliative Care. 2008, 7:10. 10.1186/1472-684X-7-10.

PPS level	Ambulation	Activity and evidence of the disease	Self-care	Intake	Conscious level
100%	Full	Normal activity and work. No evidence of the disease	Full	Normal	Full
90%	Full	Normal activity and work. Some evidence of the disease	Full	Normal	Full
80%	Full	Normal activity with the effort. Some evidence of disease	Full	Normal or reduced	Full
70%	Reduced	Unable normal job/work. Significant disease	Full	Normal or reduced	Full
60%	Reduced	Unable hobby/housework. Significant disease	Occasional assistance necessary	Normal or reduced	Full or confusion
50%	Mainly sit/lie	Unable to do any work. Extensive disease	Considerable assistance required	Normal or reduced	Full or confusion
40%	Mainly in bed	Unable to do the most activity. Extensive disease	Mainly assistance	Normal or reduced	Full or drowsy +/- confusion
30%	Totally bed bound	Unable to do any activity. Extensive disease	Total care	Normal or reduced	Full or drowsy +/- confusion
20%	Totally bed bound	Unable to do any activity. Extensive disease	Total care	Minimal to sips	Full or drowsy +/- confusion
10%	Totally bed bound	Unable to do any activity. Extensive disease	Total care	Mouth care only	Drowsy or coma +/- confusion
0%	Death	-	-	-	-
